# Extended theory of planned behavior to explain the influence mechanism of low-speed driving behavior

**DOI:** 10.1371/journal.pone.0287489

**Published:** 2023-10-13

**Authors:** Jinliang Xu, Huan Liu, Xianyong Liu, Chao Gao

**Affiliations:** 1 School of Highway, Chang’an University, Xi’an, Shaanxi, China; 2 CCCC First Highway Consultants Co., Ltd, Xi’an, Shaanxi, China; The University of Faisalabad, PAKISTAN

## Abstract

Low-speed driving is an underestimated dangerous behavior that may cause safety issues, such as speed dispersion and traffic flow bottlenecks. To investigate the influence mechanism of low-speed driving behavior, this study constructed the low-speed specific model (LSSM) by extending theory of planned behavior (TPB). The LSSM incorporated two factors, namely, risk perception and behavior habit, into the standard TPB components (attitude, subjective norm, perceived behavioral control, and behavior intention). Web-based questionnaires were used to collect data from a valid sample of 374, of which males accounted for 50%. The participants were aged from 18 to 65 years (M = 35.40, SD = 0.88). The structural equation model was applied to calculate and validate the interrelationships among the components of LSSM. Results showed that the LSSM could explain the variance in low-speed driving behavior and behavior intention by 46% and 76%, respectively. Meanwhile, attitude (β = 0.52, p < 0.001) and behavior habit (β = 0.48, p < 0.001) had the strongest positive influence and prediction power over low-speed driving behavior, respectively, whereas subjective norm (β = 0.05, p > 0.01) and perceived behavioral control (β = -0.12, p > 0.01) showed few significant in influencing the intention. LSSM also showed that people who were sensitive to driving risk perception would avoid low-speed driving behaviors and attitudes. Our findings may provide theoretical support for interventions on low-speed driving behavior.

## 1. Introduction

The discrete character of speed was always reported to be a key factor influencing accident rates [1, 2]. While the discrete character of speed is mostly caused by low-speed driving behavior, such behavior has often been overlooked. For instance, young drivers and people driving while distracted by their smartphones coincidentally drive frequently at low speed, which they perceive as a safe driving behavior, especially in highway. However, approximately 90% of collision accidents involve vehicles whose speeds did not exceed 55 km/h [3, 4]. Moreover, low-speed vehicles had been reported to cause traffic jams, thereby severely reducing the highway service level and wasting traffic resources [5–7]. These statistics highlight the need for further research into the influence mechanism of low-speed driving.

Speed selection behavior showed significant differences among individuals [8]. To explain the relationship between individual differences and speed selection, Atombo analyzed the effects of the road environment, situational factors, and individual characteristics on speed choice in simulation experiments [9]. Their regression analysis results reveal that gender, age, and driving experience all determine the perceptions and attitudes toward speed choice behavior. Specifically, males were more likely to exceed the speed limit than females [10]. Young drivers had erratic control over speed than adults [11], but young drivers with extensive driving experience can control their vehicles easily [12]. It followed that age, gender, and driving experience are not independent of one another and can hinder the prediction of low-speed driving behavior. Therefore, the Theory of planned behavior (TPB) were applied to analyze the correlation between driving behavior and psychological factors, such as attitude and perception [13, 14].

So far, the Theory of planned behavior has been successfully applied to predict various traffic violations, such as fatigue driving [15], driving while distracted by smartphones [16–18], and speeding [19, 20]. A study on speeding behavior revealed that TPB informs 47% of behavioral changes among drivers [21]. Almost one-third of driving behavior prediction studies were based on TPB. TPB constructs have also been proven to explain 40% to 70% of the variance in speeding behavior intention and 33% to 47% of the variance in self-reported speeding behavior [22–24]. The structural equation model (SEM) is often fitted with TPB to assess latent variables and their effects on the observed variables [25, 26]. Typical research that apply SEM reveals that attitude and perceived behavioral control are negatively related to speeding behavior intention, whereas subjective norms, traffic law enforcement, and awareness are positively related to speeding behavior intention [27]. The above considerations illustrate that TPB can provide theoretical support for explaining low-speed driving behavior, whereas SEM can provide technical support for quantitatively assessing such behavior. However, almost none of the studies used the theory of planned behavior to explain low-speed driving, especially in China. And it created an opportunity for this study.

This study builds and estimates a model for explaining low-speed driving behavior and assessing the influence of human factors that directly or indirectly determine such behavior. The backbone of LSSM is extended TPB. Specifically, the behavior habit and risk perception are introduced into standard TPB in conjunction with attitude, perceptual behavioral control, and subjective norm. In addition, based on the TPB survey scale and findings related to driving behavior, this study proposes a valid and reliable structure scale for measuring the abovementioned factors. The SEM is implemented to test the validity of LSSM and to assess the influence degree of potential variables.

The rest of this paper is organized as follows. Section 2 constructs the LSSM and presents the variables and model assumptions of extended TPB. Section 3 discusses the research method and data collection procedure. Section 4 highlights the results of the LSSM test and builds the LSSM estimation results. Section 5 discusses the above results, limitations of this study, and proposed interventions. Section 6 offers the key conclusions and suggestions for further research.

## 2. Theoretical approach on low-speed specific model (LSSM)

### 2.1. Extending TPB by adding personality characteristics

Although TPB has been proven to have strong applicability in practice [28], this model suffered from its static explanatory nature [29] and could not be easily applied for the reverse causation between model constructs [30]. To address such limitations, Li et al. proposed the extended TPB, which can help explain the additional variance in both intention (13%) and behavior (4%) [31, 32]. Several studies have used the special components of driving behavior to predict speed selection, which were presented in Table 1. In which, risk perception, behavior habit, law enforcement knowledge, moral norms, and sensation seeking were commonly used to predict driving behavior [33]. Moral norm represented to the particular act is morally right or wrong, with clearly defined in social rules. Such as, water-efficiency behavior is praised, while speeding is punished [34, 35]. However, low-speed driving behavior in China was not governed by social order and norms. And low-speed driving does not satisfy the desire for sensation seeking. Drivers are always eager for higher speed. It was clear that low-speed driving, as a niche group behavior, did not have the characteristics of conformity tendency. Hence, few studies on the influence mechanism of low speed driving behavior have been conducted in China. Some speeding studies provided a theoretical basis for the present study. For instance, Preece found that speeding intention is significantly predicted by the past behavior and risk perception of drivers [36]. In addition, Logan found that impulsive and profit-driven behaviors can significantly influence speed selection intention [8]. These studies illustrated that the inclusion of low-speed driving components may improve the explanations and predictions of TPB.

**Table 1:** A summary of extension variables identified in driving behavior study.

**Study**	**Topic**	**Extension variables**									**Illegal?**
		**Driver demogra-phics**	**Risk perception**	**Law enforcement knowledge**	**Moral norms**	**Behavio-r habit**	**Morml-essness**	**Sensitivity to reward/punishment**	**Conformity tendency**	**Sensation seeking**	
**Preece et al.** [36]	Speeding	√	√	-	-	√	-	-	-	-	Yes
**Chen et al.** [37]	Speeding	√	√	-	-		√	-	-	-	Yes
**Vankov et al.** [12]	Speeding	√	√	-	-	√	-	√	-	-	Yes
**Cristea et al.** [38]	Speeding	√	-	√	√	√	-	-	-	√	Yes
**Jovanović et al.** [39]	Speeding	√	-	-	√	√	-	-	-	-	Yes
**Chung et al.** [40]	Aberrant driving	√	-	-	-	√	-	-	-	-	Yes
**Yadav et al.** [33]	Drunk driving	√	√	-	√	√	-	-	√	√	Yes
**Zhenming et al.** [31]	Risky behavior	√	√	-	-	-	-	-	-	√	No
**Sadia et al.** [10]	Speed selection	√	√	-	-	-	-	-	-	-	No
**Jiang et al.** [15]	Fatigue driving	√	-	-	-	-	-	-	-	-	No
**Phuksuksakul et al.** [17]	Phone use while driving	√	√	√	-	-	-	-	-	-	No
**Qu et al.** [18]	We chat use while driving	√	-	-	√	-	-	-	-	-	No
**Yang et al.** [41]	Yielding behavior	√	√	-	-	-	-	-	-	-	No

Our LSSM was built on the work of [37], who validated the application of an extended TPB model in underestimating risk perception evaluations among 376 participants. They extended TPB by splitting its standard constructs (attitude, subjective norm, and perceived behavioral control) and by adding additional predictors, including normlessness and risk perception. Risk perception referred to people’s subjective judgment about the characteristics and severity of a particular risk. Several studies have used risk perception for predicting violations, such as speeding and distracted driving [10, 17, 42], but also included standard TPB components. Among these studies, Tang and Vankov found that risk perception significantly inhibits the red-light running behavior of electric biker users [12, 43]. Anda speeding intention study found that the behaviors of young drivers speeding at more than 60 km/h of the speed limit are significantly predicted by their risk perception [8]. However, this study specifically focused on a type of risk perception with a mediating effect. For instance, Kummeneje et. al indicated that risk perception was a crucial factor in determining the attitudes toward risk-taking behavior of cyclists [44]. Li et.al reported that risk perception of truck drivers indirectly negatively influences their intention to risky driving with the mediation of attitude toward risky driving [31].

Besides risk perception, the construct of habits has frequently been added to extend TPB. A rural road study revealed that behavior habit positively influences speeding behavior and is a strong predictor of speeding behavior in addition to intention [39]. Vankov confirmed these findings in their study on teenage speeding behavior [12]. Apart from speeding behavior, researchers have also identified behavior habit as a statistically significant predictor of violations, such as red-light running behavior and drunk driving [38, 45, 46]. Regardless of the supporting findings, some ambiguities have led to debate. Ajzen and Fishbein argued that behavior habit has a number of facets, and frequency of past behavior is a construct without explanatory value [47]. Verplanken et.al had support mightily with it. Because the strong relation between frequency of habit and behavior only reflected the temporal stability of the risky behavior [48]. That is, the corresponding behavior would be open if factors that influenced the behavior in the past remain unchanged. According to the findings on the behavior habit, Chung et.al demonstrated that the habit (habit strength measured by the self-report habit index) of speeding behavior played a crucial influence with t speeding behavior with the mediation of speeding intention [40].

The above discussions illustrated that risk perception and behavior habit have positive roles in explaining and predicting speeding behavior. However, TPB has never been applied in behavioral studies focusing on low-speed driving. To address this gap, this study incorporated two personality traits, namely, behavior habit and risk perception, into the LSSM to enhance the explanatory and predictive power of the model.

### 2.2 Model framework and assumptions

In this study, attitude (ATT) represents an individual’s positive or negative evaluation of low-speed driving behavior, subjective norm (SN) refers to the participants’ perceived social pressure to drive at low speed, and perceived behavioral control (PBC) evaluates the driver’s perceived ease of performing low-speed driving. When drivers are not confident in their driving skills, they tend to drive at speeds well below the average traffic flow velocity, even in wide road conditions. Meanwhile, behavior habit (BH) refers to the strength to which drivers are accustomed to driving at low speed in their thinking and action, and risk perception (RP) refers to the driver’s subjective judgment of the risk of low-speed driving. The following hypotheses are proposed:

Hypothesis 1 (H1). Attitude positively influences the intention to low-speed driving.
Hypothesis 2 (H2). Subjective norm positively influences the intention to low-speed driving.
Hypothesis 3 (H3). Perceived behavioral control positively influences the intention to low-speed driving.
Hypothesis 4 (H4). Risk perception negatively influences the attitude to low-speed driving.
Hypothesis 5 (H5). Risk perception negatively influences the intention to low-speed driving.
Hypothesis 6 (H6). Behavior habit positively influences the intention to low-speed driving.
Hypothesis 7 (H7). Intention to low-speed driving positively influences the low-speed driving behavior.

**Fig 1:**
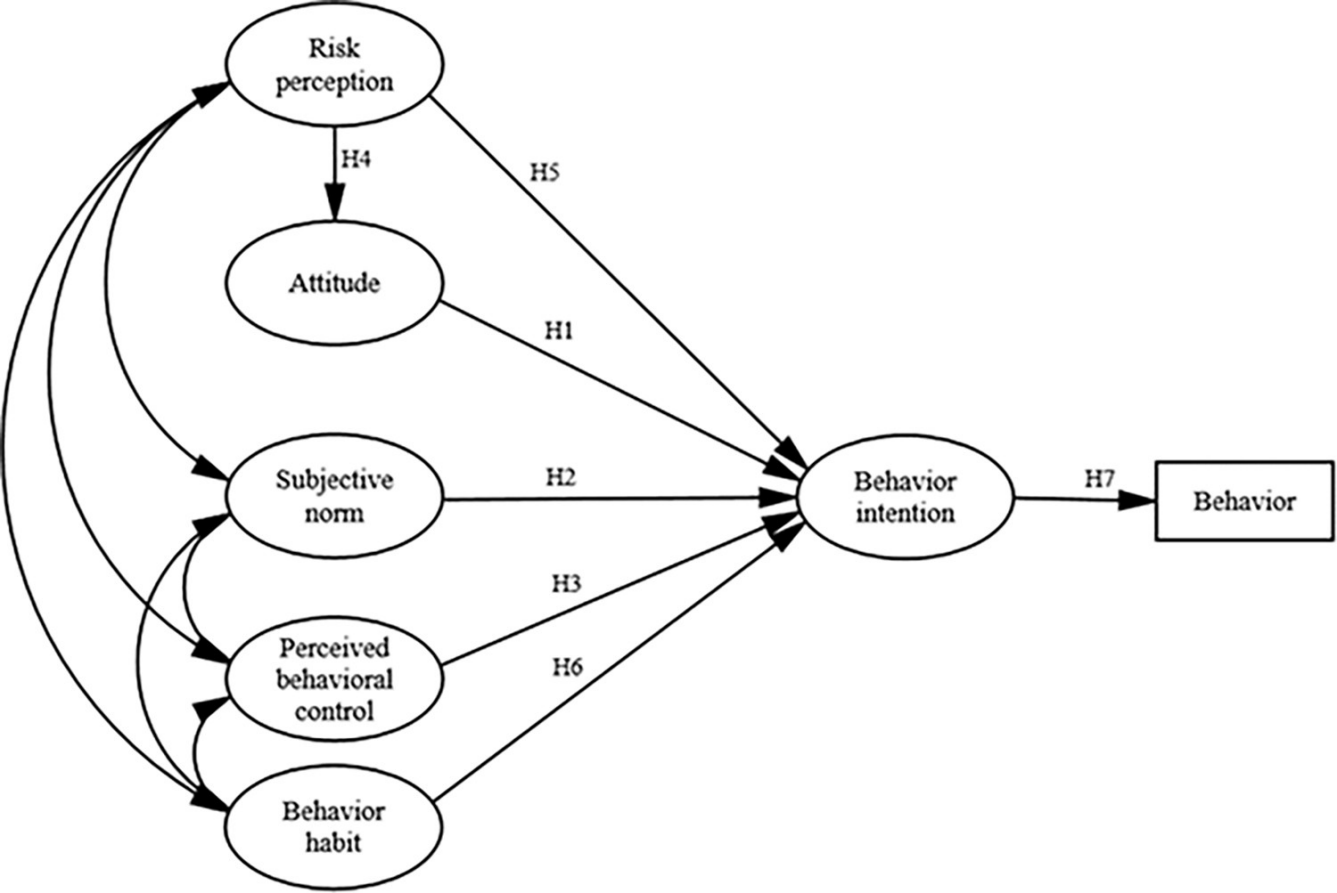
Low-speed specific model.

Based on the discussions and hypotheses presented above, this study first explored the efficacy of the standard TPB components in predicting the intention to low-speed driving. Secondly, the study identified the extension variables which may influence the drivers’ decision to engage in the act of low-speed driving. Finally, the researchers investigated the variables that play a significant role in low-speed driving. To meet these objectives, the low-speed specific model to explain the intention to low-speed driving is shown in Fig 1. And the LSSM was constructed by combining the standard TPB components, behavior habit, and risk perception. Do both standard and extended variables affect low-speed driving intention? To answer this question, the SEM was used to test the above hypotheses. Moreover, the present study examined the extent of the influence of these variables on the intention to low-speed driving by combining SEM and LSSM.

## 3. Methods

To improve the readability of the survey content, a specific explanation of low-speed driving was initially provided. In this study, low-speed driving referred to scenarios where the driving speed is lower than at least 10 km/h below the speed limit regardless of road type. Because the low-speed driving is a manifest variable, without observational variables to auxiliary measure. In this study, low-speed driving was assessed by one item (i.e., “How many times in general do you drive at a lower 10 km/h below the speed limit?”).

### 3.1. Survey design

To investigate the influence mechanism of low-speed driving behavior, this study used the causal research method. Data for this research were collected from the network survey, the Questionnaire Star online platform (https://www.wjx.cn/) was used for the survey. The network survey method has been widely used in driving behavior studies due to its low cost, high efficiency [41, 49], and reliability [50]. To further enhance the validity of the survey, a special "test question" (i.e., “Please select ’partially agree’ for this question; otherwise, your questionnaire response is deemed invalid”) was added to the questionnaire. The participants were given at least 110 s to complete the questionnaire. The failure to meet this time would prevent the participants from submitting their responses.

### 3.2. Instruments

The instruments used in this study were self-report questionnaires. Given the differences in the nature and independence of the factors, the questionnaire was divided into two parts, namely, physical factors and mental factors, as shown in Table 2.

**Table 2:** Constructs and references of survey.

Construct	Item	Category	Reference
**Physical factors**	Gender	Demographic factors	/
	Age		
	Driving age		
	Annual mileage (km)		
	Monthly income (RMB)		
	Education level		
	Insurance other than mandatory insurance		
	Accident experience	Driving experience factors	/
	Low-speed driving frequency		
**Mental factors**	Attitude	Standard TPB components	Adapted from TPB scale [12, 51]
	Subjective norm		
	Perceived behavioral control		
	Behavior intention		
	Behavior habit	Addition factors	Adapted from [12, 38, 39, 45]
	Risk perception		Adapted from [10, 17, 42]

Physical factors include demographic and driving experience factors. The demographic factors include seven items, such as gender, age, driving age, annual mileage, monthly income, education level and insurance, whereas driving experience factors included two items, such as accident experience and driving frequency related to low-speed driving. Mental factors were primarily theoretical structural measures of drivers’ low-speed intention and behavior that can be divided into 6 categories and measured on a scale from 1 to 5 (see Table 3). The standard TPB components (attitude, subjective norm, and perceived behavioral control) were measured using 12 items adapted from the TPB scale [12, 51], whereas behavior habit and risk perception were measured by 7 items adapted from the literature [10, 12, 17, 38, 39, 42, 45].

**Table 3:** Low-speed intension and behavior scale: Question and range.

Category	Item	Question	Scale (1–5)
**Attitude**	ATT1	You like to drive at a low-speed.	“Definitely disapprove” to “Definitely approve”
	ATT2	It is more pleasant for you to drive at a low-speed.	
	ATT3	You think it is safe to drive at a low-speed.	
	ATT4	You think that keeping low-speed driving will lead to traffic jam.	
**Subjective norm**	SN1	Other drivers on the road drive at a low-speed.	
	SN2	Your colleagues and friends will agree that you should drive at a low-speed.	
	SN3	Your family members often drive at a low-speed.	
	SN4	Your family members will agree that you should drive at a low-speed.	
**Perceived behavioral control**	PBC1	You can easily deal with various emergencies when you drive at a low-speed.	
	PBC2	You think it is difficult to keep driving with the traffic speed limit.	
	PBC3	You think driving at a low-speed is an easy thing to do.	
	PBC4	You are so confident in your driving skills that you can keep the low-speed driving.	
**Behavior habit**	BH1	Driving at a low-speed is something you do voluntarily	
	BH2	You always drive at a low-speed unconsciously.	
	BH3	Driving at a low-speed is something you don’t think twice about doing	
	BH4	Driving at a low-speed is something you start doing before you realize it.	
**Behavior intention**	IN1	you will actively choose to drive at a low-speed in the future.	
	IN2	You will not actively choose to drive at a low-speed in the future.	
	IN3	you have the idea of driving at a low-speed while driving in the future.	
**Risk perception**	RP1	Low-speed driving can be seriously injured.	
	RP2	Low-speed driving can cause traffic accidents	
	RP3	Low-speed driving will result in fines and adding points to your driver’s license.	

### 3.3. Ethics statement

This research was reviewed and approved by the Research Ethics Committee of Chang’an University before the experiment. And the research content strictly followed the Declaration of Helsinki. All participants have signed the informed consent.

### 3.4. Participants

The participants all had a driving license and included both professional and non-professional drivers. A total of 414 participants answered the questionnaires between April 15, 2022 and June 27, 2022. A total of 40 questionnaires were deemed invalid and were thus excluded from this study, leaving 374 valid questionnaires (90.34% of the total sample) for the analysis. This sample met the sample size requirements of the observed variables in the SEM [52]. Specifically, a sample of 374 participants (of which 50% are male) aged between 18 and 65 years (M = 35.40, SD = 0.88) drove almost 30000 km per year.

### 3.5. Procedure

The survey was divided into pre-survey and formal survey stages. At the pre-survey stage, several experts in transportation were invited to participate in a scale professionalism assessment meeting to conduct expert validity tests on the questionnaire. Meanwhile, approximately 20 participants with undergraduate degrees or higher were invited to participate in the survey and offered their suggestions to assist in refining the survey items, improved the normalization and comprehensibility of the questionnaire, and remove any ambiguous questions.

At the formal survey stage, the participants were openly recruited through various social media, such as WeChat and QQ. Each participant was informed in advance about the purpose of the survey, the types of questions, and the potential risks (e.g., leaking information and inducing consumption if the advertisement link of the Questionnaire Star online platform was mistakenly clicked). They were also informed about the confidentiality and anonymity of their responses. The formal survey was completed in an average time of 115 s. The participants also joined a lottery for a gift card or cash prize worth 20 CNY. The questionnaire access channel was set to be accessed only once per user. At the beginning of the formal survey, the participants were asked to sign an informed consent.

### 3.6. Data transformation

The measures for attitude, perceived behavioral control, and behavior intention were reverse scored, with higher scores indicating a greater agreement with a particular construct. To assess the relationship between attitude, perceived behavioral control, behavior intention, and low-speed driving, the score of ATT4, PBC2, and IN2 should be reversed to weaken the influence of the subject’s mindset on the results and to improve the validity of the questionnaire.

### 3.7. Data analysis

Outlier tests were performed on 374 samples, and results showed that outliers do not significantly affect the analysis. On the basis of the data characteristics, the data processing was divided into the following parts:

1. Reliability and validity analyses
  To eliminate non-compliant score items, the questionnaire was subjected to reliability and validity analyses using SPSS 25.0. The reliability was tested by calculating the internal consistency reliability coefficient (Cronbach’s α) of the scale, whereas the validity was tested based on Kaiser-Meyer-Olkin (KMO) and Bartlett’s sphericity values.
2. Pearson correlation analysis
  Pearson correlation analysis was conducted to determine the correlation among the variables in LSSM (attitude, subjective norm, perceived behavioral control, risk perception, behavior habit, and behavior intention) and to check the validity of the data.
3. Structural equation model
  To verify the validity of the LSSM and assess the influence degree of variables on low-speed driving, SEM was performed using maximum likelihood estimation on AMOS 23.0 (International Business Machines Corporation, New York, America). First, the confirmatory factor analysis was used to verify the validity and reliability of the potential variables in LSSM [53]. The confirmatory factors include convergent validity and discriminant validity. Composite reliability (CR) and average variance extracted (AVE) were selected for the convergent validity to evaluate the degree of consistency among multiple observed variables for the same latent variable. The selected values in this study were greater than or equal to 0.7 for composite reliability and greater than 0.5 for AVE. And the values of AVE between 0.36 and 0.5 were also acceptable. The standardized factor loadings of the observed variables should be greater than 0.7 to ensure the convergent validity of each latent variable measure [54, 55]. Discriminant validity was assessed by the square root of AVE. Each latent variable has acceptable discriminant validity when the square root of its AVE is greater than the correlation between this variable and the other latent variables in the model.
  Following SEM application studies, the standardized residuals (SRMR), comparative fit index (CFI), Tucker–Lewis index (TLI), and root-mean-squared error of approximation (RMSEA) were used to determine the overall fitness of the model. The normed fit index (NFI) and goodness-of-fit index (GFI) were then used to compare the hypothetical and independent theoretical models. The chi-squared freedom ratio (*χ*^2^/*df*) was used to represent the weighted analysis of the model freedom ratio. The selected values in this study were all less than 3.0 for *χ*^2^/*df*, less than 0.08 for the standardized residuals and root-mean-squared error of approximation, and greater than 0.8 for Tucker–Lewis index, comparative fit index, normed fit index, and goodness-of-fit index [56, 57].
4. ANOVA analysis
  To understand the effects of individual differences, a one-way ANOVA was used to assess the demographic factors and driving experience.

## 4. Results

### 4.1. Descriptive statistics

The descriptive statistical characteristics are presented in Tables 4 and 5 along with additional details about the demographic, driving experience data, and LSSM measures data.

**Table 4:** Descriptive statistics of the LSSM measures.

Items	Responses in % (1: Definitely disapprove; 5: Definitely approve)					Mean (SD)
	1	2	3	4	5	
**Attitude**						
**ATT1**	32.35	29.68	20.86	12.57	4.55	2.27(1.17)
**ATT2**	27.27	36.63	26.74	8.56	0.80	2.19(0.96)
**ATT3**	15.51	23.53	24.06	21.66	15.24	2.98(1.30)
**ATT4**	22.46	43.58	20.32	8.29	5.35	2.30(1.07)
**Subjective norm**						
**SN1**	7.22	15.78	18.72	43.85	14.44	3.43(1.13)
**SN2**	24.33	31.28	21.93	18.18	4.28	2.47(1.17)
**SN3**	27.27	37.70	17.38	12.57	5.08	2.30(1.15)
**SN4**	11.23	43.58	25.40	16.04	3.74	2.58(1.01)
**Perceived behavioral control**						
**PBC1**	10.16	16.58	19.52	30.21	23.53	3.40(1.29)
**PBC2**	30.21	36.36	19.52	11.76	2.14	2.19(1.06)
**PBC3**	29.14	27.27	21.93	14.97	6.68	2.43(1.24)
**PBC4**	12.57	8.02	27.81	33.16	18.45	3.37(1.23)
**Behavior habit**						
**BH1**	13.90	40.11	22.73	13.90	9.36	2.65(1.16)
**BH2**	5.61	15.51	15.78	45.99	17.11	3.53(1.11)
**BH3**	29.95	29.95	20.59	14.44	5.08	2.35(1.19)
**BH4**	23.80	29.95	20.59	19.25	6.42	2.55(1.22)
**Behavior intention**						
**IN1**	44.92	25.94	16.31	11.76	1.07	1.98(1.09)
**IN2**	29.14	34.76	24.33	7.22	4.55	2.23(1.09)
**IN3**	25.40	28.61	22.19	18.72	5.08	2.49(1.20)
**Risk perception**						
**RP1**	5.08	5.88	27.81	34.76	26.47	3.72(1.08)
**RP2**	4.81	5.08	19.79	41.18	29.14	3.85(1.05)
**RP3**	4.55	6.68	30.75	28.88	29.14	3.71(1.09)

**Table 5:** Descriptive statistics of the participants.

Variables	Description	Frequency	Percentage (%)
**Gender**	Male	187	50.00
	Female	187	50.00
**Age group**	18–30	169	45.20
	31–40	117	31.30
	41–50	75	20.10
	51–60	12	3.20
	≥60	1	0.30
**Driving age group**	≤1year	38	10.20
	2–5 years	106	28.30
	6–10 years	136	36.40
	≥11 years	94	25.10
**Miles driven per year (km)**	<10,000	62	16.60
	10,000–20,000	121	32.40
	20,000–40,000	81	21.70
	40,000–60,000	67	17.90
	≥60,000	43	11.50
**Monthly income (RMB)**	≤5,000	79	21.10
	5,000–10,000	202	54.00
	≥10,000	93	24.90
**Education level**	Lower secondary or below	20	5.30
	Secondary education	137	36.60
	Tertiary education	217	58.00
**Any insurance other than the mandatory car insurance**	Yes	241	64.40
	No	133	35.60
**Accident experience**	Yes	134	35.80
	No	240	64.20
**Frequency of driving below the speed limit by more than 10km/h when conditions permit**	Never	58	15.50
	Rarely drive at low-speed	131	35.00
	Sometimes/occasionally dive at low-speed	142	38.00
	Often dive at low-speed	42	11.20
	Always dive at low-speed	1	0.30

The distribution of each component of the LSSM, as well as the mean and standard deviation of each component, were shown in Table 4. Further, Table 5 reported that males account for 50% of the sample, and most respondents (45.2%) belong to the 18–30 years age group. Compared with the gender and age composition of Chinese motor vehicle drivers in 2022, the sample structure can represent a typical group of Chinese drivers in this study. Moreover, most of these drivers have a driving experience of more than 6 years and drive 27,500 km per year. The majority of the respondents are middle income earners (i.e., 54.0% of the sample earn 5,000 RMB to 10,000 RMB every month). In terms of education, more than 50% of the sample have completed tertiary education or above undergraduate education. Most of the participants have also purchased insurance other than mandatory car insurance. More than half of them have no accident experience, and 84.5% of the participants have reported driving at low speeds. These statistical results confirm that the sample covers different demographic characteristics and driving experiences, hence validating its excellent structure.

### 4.2. Reliability and validity analysis

To eliminate non-compliant score items, the reliability and validity of the questionnaire were tested on SPSS 25.0. The test results are shown in Table 6.

**Table 6:** Reliability and validity tests of the scale.

Variable	Item	Corrected item-total correlation	Cronbach’s α after deletion of items	Cronbach’s α	KMO	Sig.
**ATT**	ATT1	0.629	0.644	0.756	0.874	0.00
	ATT2	0.577	0.683			
	ATT3	0.516	0.718			
	ATT4	0.489	0.723			
**SN**	SN1	0.347	0.815	0.762		
	SN2	0.586	0.691			
	SN3	0.659	0.649			
	SN4	0.685	0.645			
**PBC**	PBC1	0.528	0.629	0.711		
	PBC2	0.280	0.760			
	PBC3	0.541	0.621			
	PBC4	0.660	0.542			
**RP**	RP1	0.585	0.818	0.82		
	RP2	0.739	0.685			
	RP3	0.701	0.723			
**BH**	BH1	0.521	0.731	0.764		
	BH2	0.441	0.759			
	BH3	0.679	0.643			
	BH4	0.622	0.676			
**IN**	IN1	0.721	0.704	0.819		
	IN2	0.613	0.809			
	IN3	0.692	0.734			

Note: ATT = attitude; SN = subjective norm; PBC = perceptual behavior control; RP = risk perception; BH = behavior habit; IN = low-speed driving intention; KMO = Kaiser–Meyer–Olkin.

As shown in the Table 6, all correlation coefficients of the corrected items exceed 0.7, thereby suggesting that the questionnaire has high reliability. Except for SN1 and PBC2, the Cronbach’s α of items after deleting certain items were lower than the overall reliability coefficients. Therefore, SN1 and PBC2 were deleted to meet the scale reliability requirements. The questionnaire also showed good validity as confirmed by its KMO (0.874) and Bartlett’s sphericity values (sig<0.05).

### 4.3. Correlation analysis

To assess the validity of the sample, Pearson correlation analysis was performed on six variables (attitude, subjective norm, perceived behavioral control, risk perception, behavior habit, and behavior intention) as shown in Table 7.

**Table 7:** Pearson correlation tests of six variables.

Variable	ATT	SN	PBC	RP	BH	IN
**ATT**	1					
**SN**	.363**	1				
**PBC**	.377**	.292**	1			
**RP**	-.329**	-0.091	-.167**	1		
**BH**	.573**	.318**	.521**	-.220**	1	
**IN**	.728**	.342**	.395**	-.194**	.688**	1

Note: ATT = attitude; SN = subjective norm; PBC = perceptual behavior control; RP = risk perception; BH = behavior habit; IN = low-speed driving intention

** Correlation significant at 1% level.

Table 7 shows that these six variables are correlated with one another. In which, the intention to low-speed driving showed significant associations with the attitude (r = 0.728, p <0.01) and behavior habit (r = 0.688, p <0.01). Therefore, SEM can be used for the subsequent analysis to further calculate the correlation among these variables.

### 4.4. Confirmatory factor analysis

Convergent validity and discriminant validity tests were performed on six latent variables to determine their accuracy. The convergent validity results are shown in Table 8.

**Table 8:** Results of convergent validity.

Construct	Item	Standardized factor loading	CR	AVE
**ATT**	ATT1	0.828	0.804	0.510
	ATT2	0.739		
	ATT3	0.653		
	ATT4	0.617		
**SN**	SN2	0.688	0.821	0.606
	SN3	0.817		
	SN4	0.823		
**PBC**	PBC1	0.626	0.764	0.522
	PBC3	0.732		
	PBC4	0.799		
**RP**	RP1	0.639	0.827	0.619
	RP2	0.886		
	RP3	0.815		
**BH**	BH1	0.607	0.794	0.495
	BH2	0.614		
	BH3	0.828		
	BH4	0.740		
**IN**	IN1	0.851	0.823	0.610
	IN2	0.681		
	IN3	0.802		

As shown in the Table 8, the standardized factor loadings of all observed variables range between 0.6 and 0.9, and the composite reliability values of the six variables are all greater than 0.7. Except for behavior habit, the mean variance-extracted AVE of the other variables are above 0.5, thereby satisfying the convergent validity requirements. The discriminant validity test results are presented in Table 9.

**Table 9:** Results of discriminant validity.

Construct	AVE	IN	BH	PBC	SN	ATT	RP
**IN**	0.610	0.781					
**BH**	0.495	0.486	0.704				
**PBC**	0.522	0.473	0.682	0.722			
**SN**	0.606	0.275	0.246	0.291	0.778		
**ATT**	0.510	0.587	0.114	0.487	0.246	0.714	
**RP**	0.619	-0.235	-0.289	-0.206	-0.084	-0.308	0.787

As shown in the Table 9, the square roots of AVE of in the six variables are all greater than the correlations between each latent variable and the other latent variables in the model, thereby satisfying the discriminant validity requirements.

### 4.5. Model verification

**Fig 2:**
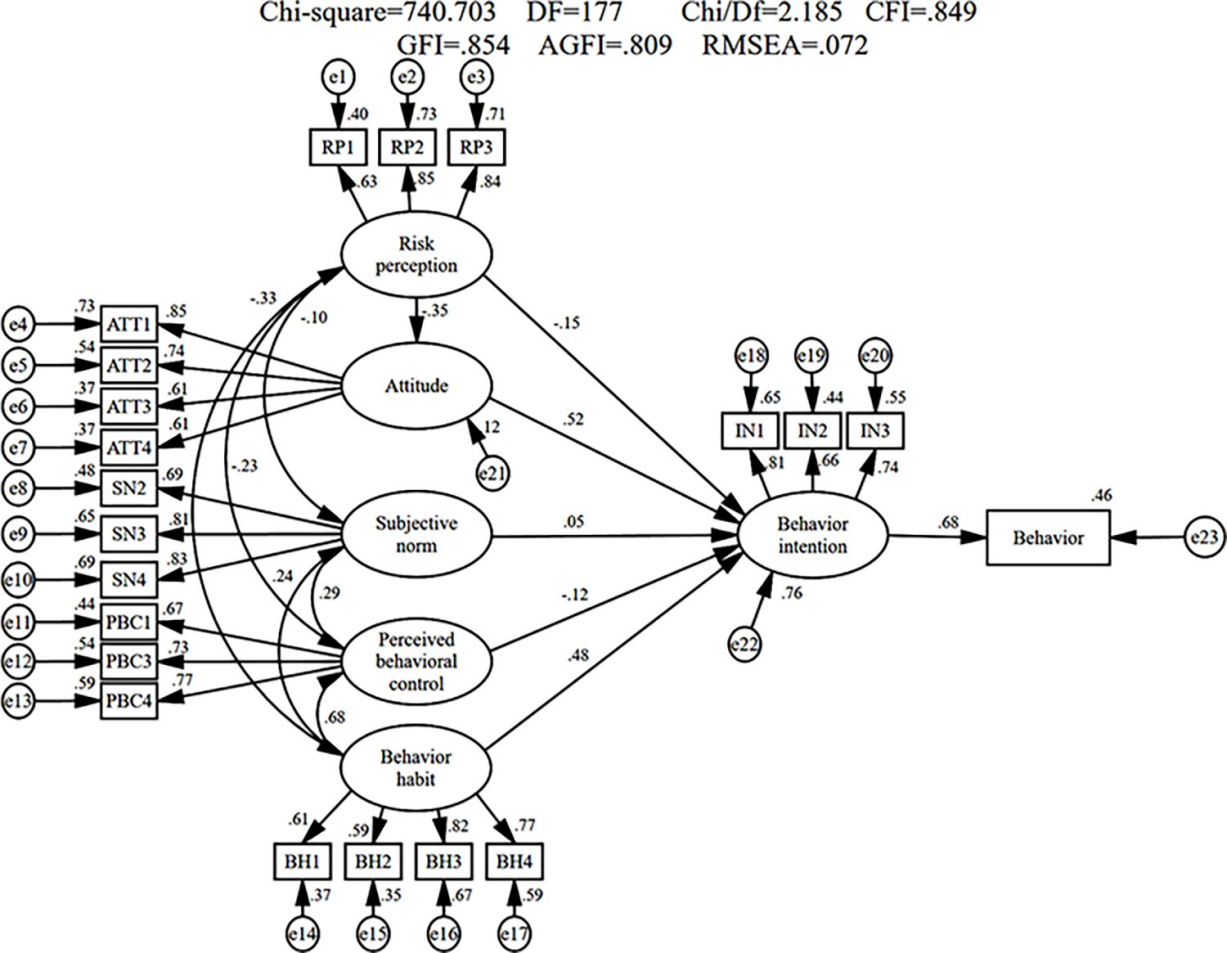
Modified model results.

To verify the effectiveness of the LSSM in predicting low-speed driving, the observed variables were fitted as input parameters for calculation and validation. The results are shown in Fig 2.

As can be seen in the Fig 2, risk perception can explain the variance in low-speed driving attitude by 12%, risk perception, attitude, subjective norm, perceived behavioral control, and behavior habit can explain the variance in low-speed driving intention by 76%, and the modified model ultimately explains 46% of the variance in low-speed driving behavior. This result indicates that LSSM has strong explanatory power for low-speed driving behavior. Specifically, attitude and behavior habit exhibit the most significant effects on behavior intention with standardized path coefficients of 0.52 and 0.48 (the correlation is significant at the 1% level). By contrast, risk perception exerts a limited negative effect on behavior intention, with a standardized path coefficient of -0.15. Meanwhile, risk perception has significant effects on attitude, with a standardized path coefficient of -0.35, thereby suggesting that this variable exerts a mediating effect. However, subjective norm and perceived behavioral control show no significant effect on behavior intention. The model was then subjected to a fitness test as shown in Table 10.

**Table 10:** Assessment of model suitability.

Model Fit Index	SRMR	CFI	TLI	RMSEA	NFI	GFI	*χ*^2^/*df*
**Evaluation Criteria**	<0.08	>0.80	>0.80	<0.08	>0.80	>0.80	<3.0
**Model Index**	0.063	0.849	0.821	0.072	0.813	0.854	2.185

As can be seen in the Table 10, all metrics meet the model evaluation criteria, thereby suggesting that the model adequately fits the data. The path coefficients (p) were then used to test the 7 hypotheses, and the results are shown in Table 11.

**Table 11:** Results of hypothesis testing.

Hypotheses	Standardized path coefficient (β)	*p*-value	Result
**H1: Attitude positively influences the intention to low-speed driving**	0.52	***	Support
**H2: Subjective norm positively influences the intention to low-speed driving**	0.05	0.181	Reject
**H3: Perceived behavioral control positively influences the intention to low-speed driving**	-0.12	0.059	Reject
**H4: Risk perception negatively influences the attitude to low-speed driving**	-0.35	***	Support
**H5: Risk perception negatively influences the intention to low-speed driving**	-0.15	**	Support
**H6: Behavior habit positively influences the intention to low-speed driving**	0.48	***	Support
**H7: Intention to low-speed driving positively influences the low-speed driving behavior**	0.68	***	Support

Note: ** Correlation significant at 1%

*** Correlation significant at 0.1%.

Attitude (β = 0.52, p<0.001) and behavior habit (β = 0.48, p<0.001) positively affect drivers’ intention of low-speed driving, thereby supporting H1 and H6. Risk perception also shows a significant negative relationship with attitude (β = -0.35, p<0.001) and low-speed driving behavior (β = -0.15, p = 0.002), thereby indicating that participants with higher risk perception have lower attitudes and frequency of low-speed driving. However, the results for the effects of subjective norm (β = -0.05, p = 0.181) and perceived behavioral control (β = -0.12, p = 0.059) on intention to low-speed driving are both insignificant, thereby indicating that subjective norm and perceived behavioral control have limited effects on low-speed driving intention. H7 is therefore supported, indicating that a stronger intention of low-speed driving (β = 0.68, p<0.001) corresponds to a higher likelihood for low-speed driving behavior to take place.

### 4.6. Effect of physical variables

The impact of physical variables on low-speed driving, such as demographic factors and accident experience, was then evaluated based on LSSM. Table 12 presents the one-way ANOVA test results.

**Table 12:** Results of one-way ANOVA.

**Construct**	**Variables**	**Comparison of differences in means**	
		**F**	**Sig.**
**Low-speed driving**	Gender	1.885	0.000
	Age group	1.628	0.167
	Annual mileage	3.154	0.014
	Monthly income (RMB)	5.050	0.007
	Education level	1.292	0.183
	Insurance other than mandatory insurance	0.296	0.000
	Accident experience	0.364	0.217

As shown in the Table 12, minimal variability (sig>0.05) can be observed among age, education level, and accident experience, whereas significant differences can be observed among gender, annual mileage, monthly income, and insurance other than mandatory insurance (sig<0.05). The results of the independent sample t-test for gender and insurance purchase reveals a difference in the mean low-speed driving scores of women than men, thereby suggesting that women are more likely to choose low-speed driving. In addition, drivers with insurance other than mandatory insurance have higher mean low-speed driving scores, thereby indicating that drivers with incomplete insurance are more likely to drive at low speed. The least significant difference results for annual mileage and monthly income show a significant positive correlation with low-speed driving, thereby suggesting that drivers with low annual mileage and monthly income intend to drive at lower speeds.

## 5. Discussion

This paper extended TPB into an LSSM consisting of split TPB constructs (attitude, subjective norm, and perceived behavioral perception), risk perception, and behavior habit. The demographic factors (gender, age, and driving age) and driving experience were treated as physical factors to explain the additional variance of low-speed driving. This study is similar to that of [37], with the difference that this study examined the ability of LSSM components to predict low-speed driving by SEM. The LSSM in this research was able to explain 76% of the variance in low-speed driving intention.

### 5.1. Recommendations and findings

The LSSM in this study helps reveal which predictors and to what extent they play a role in low-speed driving intention and behavior, thereby extending the applicability of the TPB structure to behavioral studies. Similar to the previous research [12, 37], behavior habit and risk perception were introduced into the TPB structure in this research and explained 46% variance in low-speed driving behavior. When the measures for attitude, subjective norm, and perceived behavioral control were added, their significant contributions confirmed the findings of previous studies [15–18, 22–24, 58] that about 20% to 70% of the variance in driving violation intention can be explained by extended TPB. Attitude emerged as the strongest predictor of low-speed driving intention in this paper, which also appeared for speeding and red-light running [45, 49]. This study also highlighted that those drivers who enjoy and feel safe at low speeds have stronger intention to drive at such speeds. However, most studies on speeding have reported that drivers are more likely to drive at higher speeds when no restrictions are observed, such as on small curve sections [20, 27]. When the objectives of the study are contradictory, the demographic differences in the characteristics of various driving groups need to be considered. A comparative analysis in this research revealed that women, especially those with mandatory insurance only, have a higher frequency of low-speed driving behavior. Small annual mileage and low monthly income contributed to these results. However, the opposite is true for women in the speeding driving group of previous studies. These findings indicate that targeting drivers’ low-speed driving attitudes may be a useful strategy to reduce the frequency of road traffic jams.

Unlike previous speeding research [39], neither subjective norm nor perceived behavioral control significantly affected the intention to drive at low speeds in this study. The results for subjective norm suggest that drivers do not pay attention to the opinions of their friends and family about their low-speed driving. One explanation for such finding may be that the questionnaire measures are difficult to accurately describe the concept of subjective norms, that is, the influence of society on individual behavior cannot be measured directly based on whether or not one is obedient to the will of others [59]. Another reason is that speeding behavior have motives other than low-speed behavior, and subjective norm is governed by the source of motivation [60]. Previous speeding behavior research has reported that motivation arises from rewards and punishments for certain behavior. Preceding studies also pointed out that subjective norm was moderated by certain factors, such as attitude and perceived behavioral control, which had significant mediating effects [21, 61]. Compared with speeding behavior, low-speed driving behavior in China is less regulated. Therefore, the motivation for low-speed driving is not obvious. Therefore, intervention programs should focus on establishing mandatory and subjective norms that contribute to low-speed driving behavior. Increasing public awareness about low-speed driving rules can also be a useful lever to change low-speed driving intention.

The finding that perceptual behavioral control does not directly affect low-speed driving behavior contrasts the results of previous driving behavior research [37, 58, 62]. As a predictor of behavior intention, perceived behavioral control is easily overestimated. This spuriously strong correlation may be caused by measurement redundancy and is not indicative of actual causality [63]. Many studies have also confirmed the controversial role of perceived behavioral control as a predictor of behavior intention [23, 38, 64, 65]. It has a two-layered definition, including perceived self-efficacy (the perceived ability to perform actions) and perceived controllability (the perceived control over behavioral performance) [51]. However, the TPB structure vaguely defines the concept of perceived behavioral control [39], and the participants are unable to understand the correct meaning. Nevertheless, further research should assess the causal relationship between perceived behavioral control and low-speed driving intention. Forward emphasized the influence of perceived behavioral control by showing that when drivers unconsciously change their driving practices [66], they start to resist change because they are comfortable with driving tasks that are consistent with their perceived workload (perceived self-efficacy) [67]. Therefore, the related interventions should be supplemented by a modulation of perceived behavioral control.

Past behavior is generally accepted as a predictor of future behavior and intention [68], hence explaining why behavior habit should be introduced to predict low-speed driving. Behavior habit emerges as a strong predictor in this study, and similar findings have been reported in speeding behavior prediction research [12, 23, 39]. However, unlike previous research, this study does not treat behavior habit as an independent factor, which is correlated with risk perception and subjective norm. The reason may be that behavior habit is conceptualized in this paper as the intensity of past behavior, that is, the interference of past behavioral habits on the driver’s cognition. Such difference leads to an inconsistency between behavior habit and the results of previous studies that analyze the frequency of past behavior [21, 23, 38, 66]. Following these discussions, more stringent speed control strategies and penalties should be formulated from the perspective of past behavior intensity. Moderating behavior habit can also be the key to changing the deeply ingrained behavioral practices.

Negative influences were delivered by risk perception in explaining low-speed driving intention and behavior. Drivers with high risk perception tend to be unwilling to take risks and drive at low speeds. Similar findings have been reported in risky driving behavior research [12, 43, 69]. Despite the importance of risk perception in safe driving, it had a weak direct predictive strength for low-speed driving intention in this study. Logan reported the significant mediating effect of risk perception in predicting speeding intention [8]. However, in this study, risk perception is negatively influenced by behavior habit and perceived behavioral control, thus showing a significant mediating effect. This finding might be ascribed to the definition of risk perception. Moreover, this study explores the severity of the risk of low-speed driving without considering the dimensions of probability, worry, and unsafety in risk perception [31]. Therefore, given the poor awareness of drivers about the seriousness of risks, the publicity about the seriousness of low-speed driving risks should be strengthened. Moreover, the breadth and depth of research on risk perception factors should be improved.

### 5.2. Strengths and limitations

This study confirmed the applicability of LSSM in exploring low-speed driving behavior and underscored the strong predictive effect of attitude and behavior habit. In line with [37], this study further proved that standard TPB components are useful in understanding low-speed driving intention. The results also highlighted the importance of considering the intensity of behavior habit to further understand and counteract the intention to drive at low speeds. This study also confirmed risk perception as a mediating factor that relates to attitude. These findings revealed that understanding more about the drivers’ characteristics of low-speed driving is crucial in designing interventions to change people’s behavior through education and social awareness campaigns. And intervention programs should focus on different behavior habit groups according to their levels of risk perception. Moreover, this study could help regulate driving behavior, such as changing people’s attitude in low-speed driving, correcting the relevant social norms, increasing their safety awareness, etc. to enhance traffic safety.

Some limitations of this study should be acknowledged. First, the sample involved middle-aged drivers with driving ages ranging from 5 to 10 years. However, low-speed driving intention should be investigated more thoroughly while considering the differences between driver age and driving age [70] given that novice drivers are known to have poor risk perception and are widely implicated in collisions [12]. And the web survey approach has limitations for age diversity. The vast majority of older adults were less involved with the Internet, but they may be a key group for low-speed driving. Future surveys should increase the sampling approach to take this group into account. Second, low-speed driving behavior intention is affected by multi-dimensional factors, including road conditions, traffic flow saturation, and passenger pressure [4, 71], which may also affect the speed selection behavior of drivers. However, similar to other studies, covering all potential factors under limited conditions is impossible. Therefore, future studies should try expanding the research scope to explain the impact of road conditions and traffic flow characteristics on low-speed driving intention. Third, the web-based survey results were originated from driver subjective perception of drivers as to what they would do in a hypothetical scenario, rather than what they would have done in the laboratory simulation or a real-world setting. Lack of data was to support whether survey data was consistent with actual behavior. Future research suggests a combination of subjective perceptions and objective performance for comprehensive analysis. Forth, the absence of a real-time measurement of follow-up behavior is a major issue for this study. Although previous studies have confirmed the validity of TPB in predicting driving behavior [49, 72], researchers must keep in mind that driving behavior is uncontrollable. Illegal driving that is undisturbed by past behavior and intention is also critical in ensuring traffic safety.

## 6. Conclusions

The present study conducted an examination to investigate the influence mechanism of low-speed driving behavior using the extended theory of planned behavior. According to a systematic survey of related research at home and abroad, this study is the first to investigate low-speed driving intention and behavior via a questionnaire survey in China, and the first to quantitatively assess low-speed driving by extended TPB.

1. The attitude and behavior habit were the strong predictions of low-speed driving intention. Accordingly, attitude and behavior habit clearly have more important implications for understanding low-speed driving. Safety education in low-speed driving should focus on groups with high scores for attitude and behavior, particularly the women, underinsured, and low-income groups.
2. The subjective norm, and perceived behavioral control significantly and positively influence the intention to drive at low speeds. The lack of relevant regulations and penalties for low-speed driving behaviors may also hinder drivers’ risk perception and active compliance with speed regulations. Therefore, future research should investigate the roles played by subjective norm and risk perception.
3. The risk perception showed an inhibitory effect on low-speed driving intention. As expected, the extended TPB that incorporates risk perception and behavioral habit showed a different pattern of correlation with low-speed driving intention. However, the uneven composition of annual miles driven, income level, and insurance status may compromise the generalizability of this finding. Therefore, the publicity about the seriousness of low-speed driving risks should be strengthened. And the breadth and depth of research on risk perception factors should be improved.

In conclusion, this research offers more suggestions as to what researchers might want to focus on the low-speed driving. Whereas the present study also had its limitations, e.g. lack of actual behavior data. Future research might find benefit in catching real driving data which would allow an objective assessment of low-speed driving behavior.

## Supporting information

S1 Text Certification of the ethical review for the experiment. https://figshare.com/articles/figure/Certification_of_the_ethical_review_for_the_experiment/21707582.
(PDF)

S2 Text A blank copy of informed consent. https://figshare.com/articles/figure/A_blank_copy_of_informed_consent/21707591.
(PDF)
