# Supplementary material for: Extended theory of planned behavior to explain the influence mechanism of low-speed driving behavior
Source: PLoS One. 2023 Oct 13;18(10):e0287489. doi: 10.1371/journal.pone.0287489 (PMC10575494; doi:10.1371/journal.pone.0287489)
Supplement: S1 Text — https://figshare.com/articles/figure/Certification_of_the_ethical_review_for_the_experiment/21707582. (PDF) [file pone.0287489.s001.pdf]

有关山东省交通运输厅科研研究项目涉及伦理问题及处理方式的申明

本人从事的“基于拓展计划行为理论解释低速驾驶行为影响机制”课题拟申请山东省交通运输厅科研研究项目，其研究内容涉及来源人的生物样品、私人疾病信息及生命信息等。

该课题前期研究内容严格遵循《赫尔辛基宣言》、世界卫生组织与国际医学科学组织理事会共同制定的《涉及人的生物医学研究国际伦理准则》以及山东省交通运输厅有关规定进行研究工作。在本项目的实施过程中，将严格做好知情同意、处理方案与可能的补偿方式，保证样品来源人的个人信息、医疗信息不被公开披露，在法律允许范围内尽一切努力保护样品来源者的个人医疗资料、疾病信息、生命信息和基因信息的隐私。本研究的后续研究也将继续遵循上述原则与相关规定，并接受审查与监督。

特此申明。

项目负责人：[illegible]

2022 年 4 月 11 日

单位审核意见：

[illegible] 所做“基于拓展计划行为理论解释低速驾驶行为影响机制”课题涉及的伦理问题及处理方式符合国内外相关机构的规定，同意其开展该课题研究内容的实验工作。

长安大学公路学院

2022 年 4 月 11 日
