# Supplementary material for: Extended theory of planned behavior to explain the influence mechanism of low-speed driving behavior
Source: PLoS One. 2023 Oct 13;18(10):e0287489. doi: 10.1371/journal.pone.0287489 (PMC10575494; doi:10.1371/journal.pone.0287489)
Supplement: S2 Text — https://figshare.com/articles/figure/A_blank_copy_of_informed_consent/21707591. (PDF) [file pone.0287489.s002.pdf]

《基于扩展计划行为理论的驾驶员低速驾驶影响机制》

受试者知情同意书

尊敬的受试者：

您将参加一项科研试验。本须知提供给您一些信息以帮助您决定是否参与此次试验。请您仔细阅读，如有疑问请向负责本试验的研究者提出。

您参与的本项试验是自愿的。本次研究符合《赫尔辛基宣言》的原则。

试验目的：分析驾驶员在驾驶过程中低速驾驶行为的影响因素及其影响程度。

试验对象：身体健康、有驾驶执照的驾驶员。

试验过程：如果您同意参与这项试验，您需要在网络上填写一份《驾驶员低速驾驶行为认知特性调查问卷》，问卷内容包括人口统计学信息和驾驶员低速驾驶认知量表题。该调查采取匿名做答，不收集个人隐私，数据仅作为学术研究使用，不会泄露任何个人信息，请放心填写。

您可随时了解与本研究有关的信息资料和研究进展，如果您有与本研究相关的问题，或与关于本研究参加者权益方面的问题，您可以通过电话 13324532755 与试验员刘欢联系。

受试者声明：

我已阅读了本知情同意书，且已与本试验的研究者详细讨论并了解本研究的目的、对象、过程。在仔细阅读以上有关说明后，经过充分时间的考虑，我自愿成为此项研究的受试者，积极配合研究人员进行本项试验。

受试者签名：

联系电话：　　　　　　　　　　　　　　日期：

研究者签名：

联系电话：　　　　　　　　　　　　　　日期：
